# Revisiting Species Identification within the Enterobacter cloacae Complex by Matrix-Assisted Laser Desorption Ionization–Time of Flight Mass Spectrometry

**DOI:** 10.1128/spectrum.00661-21

**Published:** 2021-08-11

**Authors:** Alexandre Godmer, Yahia Benzerara, Anne Cécile Normand, Nicolas Veziris, Salah Gallah, Catherine Eckert, Philipe Morand, Renaud Piarroux, Alexandra Aubry

**Affiliations:** a Département de Bactériologie, Hôpital Saint-Antoine, AP-HP, Sorbonne Université, Paris, France; b Centre d’Immunologie et des Maladies Infectieuses, INSERM, U1135, Sorbonne Université, Paris, France; c Laboratoire de Bactériologie-Hygiène, Hôpital Pitié-Salpêtrière, AP-HP, Sorbonne Université, Paris, France; d Laboratoire de Parasitologie-Mycologie, Hôpital Pitié Salpêtrière, AP-HP, Sorbonne Université, Paris, France; e Département de Bactériologie, Hôpital Cochin, AP-HP, Centre-Université de Paris, Paris, France; f Institut Pierre Louis d'Epidémiologie et de Santé Publique, INSERM, UMR-S 1136, Sorbonne Université, Paris, France; Montefiore Medical Center and Albert Einstein College of Medicine

**Keywords:** MALDI-TOF MS, *Enterobacter cloacae* complex, *Enterobacter hormaechei*, mass spectrometry, *Enterobacter*

## Abstract

Matrix-assisted laser desorption ionization–time of flight mass spectrometry (MALDI-TOF MS) is commonly used by clinical microbiology laboratories to identify pathogens, despite some limitations of the technique. The Enterobacter cloacae complex (ECC) taxonomy has recently been expanded, leading to uncertain identification of some species within the ECC when commercial MALDI-TOF MS is used. This technique is especially unsuited in the case of *E. hormaechei*, the main species responsible for infections and one of the most prone, within the ECC, to acquire antibiotic resistance. Hence, rapid and reliable identification at the species level could improve patient management. Here, we evaluated the performance of the Bruker Microflex MALDI-TOF MS instrument to identify ECC isolates using two databases and algorithms in comparison to the *hsp60* gene sequencing reference method: the Bruker database included in the MALDI Biotyper software and an extensive online database coupled to an original Mass Spectrometric Identification (MSI) algorithm. Among a panel of 94 ECC isolates tested in triplicate, the online database coupled to MSI software allowed the highest rate of identification at the species level (92%) compared to the MALDI Biotyper database (25%), especially for the species *E. hormaechei* (97% *versus* 20%). We show that by creating a database of MALDI-TOF reference spectral profiles with a high number of representatives associated with the performant MSI software, we were able to substantially improve the identification of the E. cloacae complex members, with only 8% of isolates misidentified at the species level. This online database is available through a free online MSI application (https://msi.happy-dev.fr/).

**IMPORTANCE** Creation of a database of MALDI-TOF reference spectral profiles with a high number of representatives associated with the performant MSI software enables substantial improvement in identification of E. cloacae complex members. Moreover, this online database is available through a free online MSI application (https://msi.happy-dev.fr/).

## INTRODUCTION

The Enterobacter cloacae complex (ECC) is a polyphyletic group of species responsible for a wide variety of infections, especially nosocomial infections. These opportunistic pathogens are present in the environment (e.g., in soil and sewage) and in the commensal enteric flora of humans and animals (1).

The taxonomy of the ECC has greatly evolved between 1980 and 2021, with numerous rearrangements. In 2005, six pheno- and genotypically delineated species were taken to form the so-called ECC: Enterobacter hormaechei (2), Enterobacter asburiae (3), Enterobacter cloacae and Enterobacter dissolvens (3), Enterobacter kobei (4), and Enterobacter nimipressuralis (3, 5).

Since the sequencing of 16S rRNA is insufficient to accurately discriminate among the various ECC members, Hoffmann et al. proposed sequencing of the 60-kDa heat shock protein gene (*hsp60*) as reference method for the identification of ECC species (5). This allowed the identification of 12 ECC clusters (I to XII) (5), but only 13% of the 208 studied strains clustered with preliminarily known species, while the majority of clinical strains were found in only two clusters: i.e., VI and VIII. These latter clusters were located near the *E. hormaechei* clade, with no type strain representative. After further characterization by conventional taxonomic tests and whole-genome sequencing (WGS), *E. ludwigii* (6), *E. hormaechei* subsp. *oharae*, *E. hormaechei* subsp. *steigerwaltii* (7), and *E. bugandensis* (8) species were assigned to clusters V, VI, VIII, and IX, respectively (6–9), and *E. dissolvens* was assigned to a subspecies of E. cloacae (10).

Recently, *E. hormaechei* subsp. *hoffmannii* and *E. roggenkampii* were proposed as names for the orphan Hoffmann clusters III and IV, respectively, (11). Cluster X has been excluded from the ECC since it was reassigned to the *Lelliottia* genus (12).

In 2018, a first global exploration of ECC phylogeny using public WGS data was performed (11). This *in silico* research, based on the average nucleotide identity (ANI) of 1,249 NCBI RefSeq Enterobacter-labeled genomes, revealed a total of 22 clades (named A to V) spanning the 11 Hoffmann clusters. Seven of the proposed clades did not have representative type strains and make up potential new species (K, L, N, O, P, S, and T). Further analysis resulted in the deposition of a clinical type strain for each of the L, N, and T clades, named *E. chengduensis* (13), *E. sichuanensis*, and *E. chuandaensis* (14), respectively (11).

Some studies have suggested a strong link between antibiotic resistance mechanisms (15, 16), infectious tropisms (11, 17–19), or virulence factors (20, 21) and the species *E. hormaechei*. An overall genome-related index suggested that *E. hormaechei* contains at least five subspecies (clades A to E), including *oharae*, *steigerwaltii*, *hormaechei*, *hoffmannii*, and *xianfangensis* (9), as well as maybe clade S (11), while potentially different pathogenicities may require an effective identification method, and molecular methods are not suitable for daily practice in medical laboratories.

The objective of this study was to improve ECC species identification using matrix-assisted laser desorption ionization–time of flight mass spectrometry (MALDI-TOF MS), with *hsp60* gene sequencing as the reference method. Using a panel of isolates from four different hospitals, we compared the performance of two databases and algorithms: the Bruker database coupled to the MALDI Biotyper software *versus* an extensive online database coupled to an original Mass Spectrometric Identification (MSI) algorithm (https://msi.happy-dev.fr/). This online database is available through a free online MSI application.

## RESULTS

### Comparative species identification with the MALDI Biotyper and MSI systems.

Sequencing of *hsp60* segments allowed correct species or subspecies identification for 99% (133/134) of the clinical isolates. The only isolate having a dissimilarity score of >2% between its *hsp60* sequence and that of type strain sequences (Table 1) was identified as E. cloacae subsp. *cloacae* by WGS.

**TABLE 1:** Enterobacter isolates used for construction of the MSI online database and the tested panel

Species, subspecies, or clade	No. of isolates in^*a*^:	
	MSI online database	Tested panel collection
*E. asburiae*	5	6
*E. kobei*	4	3
*E. roggenkampii*	5	9
*E. ludwigii*	2	6
*E. hormaechei* subsp. *hoffmanni*	5	18
*E. hormaechei* subsp. *xianfangensis*	7	8
*E. hormaechei* subsp. *steigerwaltii*	7	34
*E. hormaechei* clade S	1	5
*E. bugandensis*	3	3
E. cloacae subsp. *cloacae*	2	1
E. cloacae subsp. *dissolvens*	1	1

Total	42	94

a The total number of isolates was 136 (134 clinical strains and 2 ATCC strains).

The replicate of one *E. kobei* isolate among those of the tested panel was removed because of very poor spectrum quality. The MSI software with the online database provided the highest identification rate (Table 2). Overall, 92% (259/281) *versus* 25% (71/281) of spectra were correctly identified at the species level with a kappa coefficient of 0.80 *versus* 0.19, using the MSI and MALDI Biotyper systems, respectively. The identification accuracy of MSI was superior to that of MALDI Biotyper software for 6/7 species (Table 2). The MSI software allowed accurate identification of more than 93% of the isolates of three species (*E. asburiae*, *E. kobei*, and *E*. *hormaechei*), while less than 44% were identified with the MALDI Biotyper software. The *E. hormaechei* species was correctly identified in 97% of cases with the MSI system *versus* 20% with the MALDI Biotyper system. *E. roggenkampii* was never identified with the MALDI Biotyper *versus* 66% of cases with the MSI system. The discrepancies observed between the identifications obtained by *hsp60* sequencing or using the MSI system mainly concerned spectra of *E. ludwigii* (*n* = 5) identified as *E. bugandensis* or spectra of *E. roggenkampii* (*n* = 5) identified as *E. ludwigii*, *E. kobei*, or *E. asburiae* (see Table S1 in the supplemental material).

**TABLE 2:** Correct identification rates for the different Enterobacter cloacae complex species and corresponding confidence scores obtained with the two identification systems used in this study

Species	MALDI biotyper software with Bruker database^*a*^		MSI software with online database^*a*^	
	% correct identification (n/N)	% precision (n/Ni)	% correct identification (n/N)	% precision (n/Ni)
*E. asburiae*	44 (8/18)	50 (8/16)	94 (17/18)	74 (17/23)
*E. bugandensis*	100 (9/9)	31 (9/29)	78 (7/9)	64 (7/11)
E. cloacae	50 (3/6)	2 (3/176)	83 (5/6)	100 (5/5)
*E. kobei*	25 (2/8)	25 (2/8)	100 (8/8)	50 (8/16)
*E. ludwigii*	56 (10/18)	77 (10/13)	72 (13/18)	81 (13/16)
*E. roggenkampii*	0 (0/24)	NA (0/0)	66 (16/24)	94 (16/17)
*E. hormaechei*	20 (39/198)	100 (39/39)	97 (193/198)	100 (193/193)

a “n” indicates the ∑ correct identification of the given species, “N” indicates the ∑ expected identification of the given species, and “Ni” indicates the ∑ correct and incorrect identifications of the given species. NA, not applicable.

Among the 94 isolates analyzed with the MSI system, 87% (82/94) were correctly identified from the three technical replicates, and 96% (90/94) of the isolates were correctly identified on at least one spot of the three replicates. Finally, 4% (4/94) of the isolates were never properly identified on the three spots analyzed.

Statistical log score differences seen with the MALDI Biotyper software between correct and false identification were not significant (mean ± standard deviation of 2.26 ± 0.08 *versus* 2.25 ± 0.09, respectively; *P* > 0.05). Conversely, MSI confidence scores from correct identifications were significantly higher (*P* < 0.0001) than those from false identifications (mean ± standard deviation of 57.33 ± 8.25 *versus* 50.23 ± 7.85, respectively). Analysis of the receiver operating characteristic (ROC) curve of the identifications using the MALDI Biotyper showed a maximum correct identification rate and a minimum incorrect identification rate when the log score threshold was 2.23 (see Fig. S2 and S3 in the supplemental material). In this case, the correct identification rate increased to only 29%, while nearly 36% of the species rank identifications were considered unreliable. With the MSI system, the maximum correct identification rate and the minimum incorrect identification rate were obtained for a confidence score higher than 51.9. In this case, the correct identification rate increased to 96.7%, but the rejection rate for species validation was 26%. To obtain a 95% correct identification rate, it was necessary to observe a threshold MSI score of 48 (Table S1). In this case, the species identification rate considered unreliable was only 14.6%. The species used to control the specificity of the MSI software (i.e., Escherichia coli, Klebsiella pneumoniae, and Streptococcus pneumoniae) had similarity scores below 20.

### Identification of discriminant peaks.

We found no single peak allowing discrimination between species. To unambiguously identify a particular species, it is therefore necessary to consider several specific and constant peaks (see Table S2 in the supplemental material). For example, it is necessary to detect three peaks (*m/z* = 3.845, 8.517, and 8.998) to discriminate between *E. hormaechei* and all other species. Of note, visualization of the average spectra of the different species with a simple interactive interface is available online (https://agodmer.github.io/ECC/).

## DISCUSSION

MALDI-TOF MS is increasingly used by clinical microbiology laboratories for the identification of pathogenic species, despite some limitations of this technique. In this study, we showed that by creating a database of MALDI-TOF reference spectral profiles with a significant number of representatives of different species coupled with an original algorithm and software (MSI), we were able to greatly improve the identification of ECC members, with only 8% of the isolates misidentified at the species level.

It is of medical importance to be able to identify ECC isolates at the species level since this complex is known to comprise important nosocomial pathogens, some of which are particularly prone to acquire antibiotic resistance. In a multicenter study from 2018 conducted in 10 French hospitals on 193 clinical ECC isolates from various samples (urine, respiratory specimens, blood cultures, and wounds), *E. hormaechei* represented approximately 80% of the recovered species, and it had the highest prevalence of resistance to third-generation cephalosporins (15). Additionally, two predominant subspecies of *E. hormaechei* were found to be especially prone to produce carbapanemases: i.e., *E. hormaechei* subsp*. xiangfangensis*, mostly of sequence type ST114, and *E. hormaechei* subsp*. steigerwaltii*, especially of sequence types ST90 and ST93 (16). Conversely, carbapenemase production was only rarely reported for E. cloacae and *E. roggenkampii* and never for the remaining ECC species (*E. kobei*, *E. asburiae*, and *E. bugandensis*).

The performance of the MALDI-TOF MS identification depends very strongly on the quality of the database used. ECC species are for the most part not or inadequately identified with the MALDI Biotyper (22 and see below), with, in this study, a 25% correct identification rate (Table 2). In particular, of the three most prevalent ECC species, *E. asburiae*, *E. kobei*, and *E. hormaechei*, only 22% (49/221) were identified by the Bruker database using the MALDI Biotyper software, whereas with the MSI software, more than 97% of them were identified accurately. The main clinical species of interest, *E. hormaechei*, was mostly misidentified, with only 20% correct identification by the MALDI Biotyper, while with the MSI system, 97% of species were identified correctly. The main reasons for the poor performance of the commercial system are related to the spectral profile contents of the database and are listed as follows. (i) The species *E. homaechei* is represented by a single main spectrum profile (MSP) from the Enterobacter hormaechei subsp. *hormaechei* type strain, a subspecies very rarely isolated in clinical samples (11). (ii) *E. roggenkampii*, a major clinical species is not represented. (iii) Three important clinical subspecies of *E. hormaechei* (i.e., *hoffmannii*, *steigerwaltii*, and *oharae*) are not represented in the database, making their identifications impossible. (iv) The MALDI Biotyper database falsely classifies *E. hormaechei* subsp. *xiangfangensis* as a species and not as a subspecies, implying that closely related subspecies (*hoffmannii* and *steigerwaltii*, as well as clade S) are frequently identified as “*E. xiangfangensis*,” which may confuse nonspecialists. (v) Finally, the taxon E. cloacae is represented by 14 MSPs, 11 of which were obtained from strains identified only at the genus level (e.g., DSM3264, Enterobacter sp.) or belonging to *E. hormaechei* subsp. *hoffmannii* (e.g., DSM3060) (Table S1). Thus, 173 spectra were wrongly identified as E. cloacae. Among these incorrect identifications, nearly 90% concerned the species *E. hormaechei*, suggesting that the spectra in the MALDI Biotyper database could derive from this species.

The performance of MALDI-TOF MS identification also depends on the quality of the spectra included in the database, which depends on the extraction methods used and on the spectral acquisition method. Recently, another study aimed to develop a MALDI-TOF MS method to improve identification of the ECC members (23). In that study, the identification performance at the species level was slightly better than ours (i.e., 100% *versus* 92%), which could be explained by the *modus operandi* of Wang et al. (23): (i) they extracted all strains using a time-consuming protocol that produces good-quality spectra but is not applicable in the daily routine of medical bacteriological laboratories; (ii) moreover, the acquisition of the spectra was performed using conditions (500 laser shot acquisitions) different from those recommended by the Bruker protocol and outside the CE-IVD or FDA specifications. This implies having two conditions of acquisition of the spectra, two settings of the device, and a lack of practicability that can lead to risks of errors for users. To our knowledge, this is the only study that has reported peaks discriminating between ECC species using the MALDI Biotyper explorer module (23). We also searched for discriminant peaks using the same method. In addition, we confirmed our results by extracting and processing the spectra using MALDIquant (24), a scripting module running in the R environment. Wang et al. (23) described eight specific peaks (at a threshold frequency of >97% and absent in other taxa) discriminating between *E. hormaechei* and other Enterobacter species, as well as 11 peaks common to all Enterobacter species except *E. hormaechei*. Unfortunately, none of these peaks was found to be discriminating in our study (Table S2). Our number of reference strains per species was lower and probably needs to be augmented with proteomically informative strains—especially some from other geographical locations, which the concept of the online MSI software should allow us to achieve.

At this stage, and for two reasons, we are not yet able to satisfactorily discriminate between the different *E. hormaechei* subspecies. First, our collection of reference strains belonging to the species *E. hormaechei* is incomplete. Clade S is represented by a single isolate and *E. hormaechei* subsp. *hormaechei* and subsp. *oharae* are not represented at all. Second, according to spectral analysis, it is delicate to identify all subspecies of *E. hormaechei* at first glance due to the close resemblance to spectral patterns of the subspecies. While *hsp60* sequencing is a powerful means of discriminating between most of the known species and subspecies of the complex (133/134 clinical strains in this study), one isolate had an *hsp60* sequence dissimilar (>2%) to those of the type strains. It is therefore necessary to further explore the phylogenetic diversity in the ECC, in particular by using WGS.

Due to their absence or scarcity in human clinical samples, our study was not able to include genera or species close to Enterobacter outside ECC as specificity controls (i.e., *Leclercia* or *Lelliottia*); therefore, our database should be used in case of ECC identification by the Bruker system (i.e., score of >2.00) since the latter system wrongly identified most of them. Some studies aim to explore the expressed virulence factors or clinical tropisms that could be species or subspecies specific. For example, some ECC strains demonstrate strong hemolytic and leukotoxic activity (20, 21), and some produce a type I or II Shiga-like-toxin (21, 25), whereas some strains of *E. hormaechei* contain a pathogenicity island coding for the mobilization of extracellular iron by a siderophore such as yersiniabactin (25). It would thus be interesting to carry out a rapid and robust identification of ECC subspecies, in particular to elucidate the involvement of certain taxa in different pathologies as well as the association of taxa and virulence factors. Access to the MSI identification system is open to the entire community of microbiologists. Collaboration with other laboratories should allow us to rapidly enrich our online database to further improve the identification of ECC species and also of *E. hormaechei* subspecies.

### Conclusion.

We have built a new reference spectral database allowing the identification at the species level, by MALDI-TOF MS, of most ECC taxa encountered in a clinical laboratory. Overall, the rate of correct identification with the MSI *versus* the Biotyper system was improved from 25% to 92%, with a good confidence score. Our study shows that the identification of a clinically important species (*E. hormaechei*) is correctly identified with the MSI software at 97% *versus* 20% with the MALDI Biotyper software. This is the first use of the MSI system for the identification of bacteria. Access is open to the entire scientific community, which will allow us to easily improve and enrich the database according to future advances in phylogenomics or following the observation of atypical spectral profiles. Eventually, the identification of subspecies of *E. hormaechei* will be developed, leading to deeper knowledge of the epidemiology and pathogenesis of this complex taxon.

## MATERIALS AND METHODS

### Bacterial isolates.

A collection of 134 clinical isolates belonging to seven ECC species were prospectively collected in one laboratory from four different Paris hospitals (Saint-Antoine, Tenon, Trousseau, and Cochin). These isolates were from human samples and prospectively taken from the daily workflow of the laboratory. Additionally, two reference strains, E. cloacae subsp. *dissolvens* (ATCC 23373) and *E. asburiae* (ATCC 35993), were included. All isolates were stored at −20°C (Microbank; Pro-Lab Diagnostics). Three isolates belonging to the species Klebsiella pneumoniae, Streptococcus pneumoniae, and Escherichia coli, which were not represented in the ECC database in MSI, were used as specificity controls to test the database.

### Online database.

Among the 136 isolates (clinical and ATCC strains), a total of 42 were selected (40 clinical isolates and 2 reference strains) to set up the MSI database for MALDI-TOF MS identification. The database was further uploaded into the MSI application (https://msi.happy-dev.fr/) to make it available for potential users.

### Tested panel.

The panel of isolates used to test the online and Bruker databases included the remaining 94 isolates from the collection. Table 1 shows the distribution of the isolates selected to be part of the online database and of those used as the test panel.

### DNA sequencing.

All 134 clinical isolates used in the study were identified using *hsp60* sequencing. Two PCRs were used: (i) a PCR enabling the sequencing of a 324-bp fragment of the *hsp60* gene (from positions 1218 to 1560) was applied to all the isolates following a protocol adapted from reference 5, and (ii) if the first sequencing identified the species *E. hormaechei*, a second *hsp60* fragment (nucleotides 230 to 1027) was sequenced after amplification with primers hsp60_230_F (5′-TTGCCTCTAAAGCGAACGAC-3′) and hsp60_1027_R (5′-GAATAGCGGCTTCTTCACCC-3′). DNA extraction was performed by using the InstaGene matrix (Bio-Rad, Marnes la Coquette, France) following the manufacturer’s instructions. PCRs were performed in a 50-μl final volume with 0.2 μM each primer, 2 μl of DNA, and 25 μl of Qiagen *Taq* PCR master mix. Amplification conditions were as follows: after 7 min of denaturation at 94°C, we used 35 amplification cycles of denaturation at 94°C for 1 min, annealing at 56°C for 30 s, and extension at 72°C for 30 s, with a final extension step for 5 min at 72°C. Sequence analysis was carried out using BioEdit v.7.0.5.3. Sutton et al. described 22 clades (A to V) and proposed a type strain for each clade (10). To evaluate the interclade discriminating power of the *hsp60* sequences to identify ECC clades, we determined them for all type strains (Table 3) and created a local database. Then, we constructed phylogenetic trees according to the maximum likelihood method from the alignments of the regions from positions 1218 to 1560 and 230 to 1027. Evolutionary analyses were conducted in MEGA X (26) (see Fig. S1 in the supplemental material).

**TABLE 3:** Correspondence between the nomenclatures of Sutton and Hoffmann with type or reference strains used for *hsp60* sequence-based identification

Reference/type strain name used for *hsp60* identification	Accession no.	Clade name	Nomenclature by:	
			Sutton	Hoffman
LMG27195	CP017183.1	*E. hormaechei* subsp. *xiangfangensis*	A	VI
DSM16691	NZ_CP017179.1	*E. hormaechei* subsp. *steigerwaltii*	B	VIII
DSM16687	NZ_CP017180.1	*E. hormaechei* subsp. *oharae*	C	VI
DSM14563	NZ_CP017186.1	*E. hormaechei* subsp. *Hoffmannii*	D	III
ATCC 49162	AFHR00000000	*E. hormaechei* subsp. *hormaechei*	E	VII
LMG25706	AEXB00000000	*E. mori*	F	Not reported
ATCC 13047	NC_014121.1	E. cloacae subsp. *cloacae*	G	XI
ATCC 23373	WJWQ00000000	E. cloacae subsp. *dissolvens*	H	XII
EN119	NZ_CP017279.1	*E. ludwigii*	I	V
ATCC 35953	NZ_CP011863.1	*E. asburiae*	J	I
1161ECLO	JWAU00000000	E. cloacae complex clade K	K	Not reported
GN02587	NZ_LEDN00000000.1	*E. chengduensis*	L	Not reported
DSM16690	NZ_CP017184.1	*E. roggenkampii*	M	IV
DS11005	NZ_NPNR00000000.1	*E. sichuanensis*	N	Not reported
GN05526	NZ_LVUF00000000.1	E. cloacae complex clade O	O	Not reported
624ECLO	NZ_JUZQ00000000.1	E. cloacae complex clade P	P	Not reported
DSM13645	NZ_CP017181.1	*E. kobei*	Q	II
cterbugandensis	LT992502	*E. bugandensis*	R	IX
ND22	LZEN00000000	E. cloacae complex clade S	S	Not reported
C9	MTKD00000000	*E. chuandaensis*	T	Not reported
ATCC 35316	ABWM00000000	*E. cancerogenus*	U	Not reported
ATCC BAA-2102	LXES00000000	*E. soli*	V	Not reported

The partial *hsp60* sequence of each clinical isolate was compared to the corresponding sequences of the reference strains of the 22 clades. Species or subspecies were assigned if the dissimilarity score between the *hsp60* sequence of the studied isolates and of the type strain sequences was inferior to 2%. If this was not the case, the closest identification was returned and named “proxy.”

### MALDI-TOF MS sample preparation and data acquisition. (i) Sample preparation for the online database.

Each isolate was thawed and cultured at 37°C for 18 to 24 h on Columbia blood agar (COH). A subculture was performed at 37°C for 18 to 24 h on the same medium. A single colony was suspended in 200 μl of water and vortexed. Then, 900 μl of ethanol was added. The samples were vortexed and centrifuged at 13,000 × *g* for 2 min. The supernatant was removed, and the residual ethanol was evaporated at room temperature. Then, 25 μl of 70% formic acid was added and mixed with the pellet. Finally, 25 μl of acetonitrile was added and mixed. After centrifugation at 13,000 × *g* for 2 min, the supernatant was ready to be spotted. Eight technical replicates were prepared for each isolate. Dried spots were overlaid with 1 μl of α-cyano-4-hydroxycinnamic acid (α-HCCA) in 50% acetonitrile–2.5% trifluoroacetic acid, and each spot was analyzed three times by MALDI-TOF MS.

### (ii) Sample preparation for the tested panel.

Each isolate was thawed and cultured at 37°C for 18 to 24 h on COH. A subculture was performed on COH at 37°C for 18 to 24 h. Then, a single bacterial colony was spotted onto a MALDI target plate by direct transfer. Dried spots were overlaid with 1 μl of α-HCCA in 50% acetonitrile and 2.5% trifluoroacetic acid. Three replicates corresponding to three identification spots were prepared for each isolate.

### (iii) Mass spectrum acquisition.

Mass spectra were acquired with a Microflex LT instrument (Bruker Daltonics) using the default parameters of the standardized CE-IVD method recommended by Bruker. This instrument was equipped with an N_2_ laser (λ = 377 nm) and the following parameters were used: mass range, 2,000 to 20,000 Da; ion source 1, 20 kV; ion source 2, 18.15 kV; lens, 6 kV; pulsed ion extraction, 150 ns; laser frequency, 20 Hz. An external calibration standard (Bacterial Test Standard; Bruker Daltonics) was used. FlexControl (version 3.0; Bruker Daltonics) was used for data acquisition.

### Mass spectrometry reference databases.

We used two databases for comparative identification: the Bruker reference database and the MSI online database set up for the study.

Using FlexAnalysis (version 4.2) software (Bruker Daltonics), spectra were visually analyzed. Poor-quality spectra were removed, and between 20 and 24 spectra per isolate were retained. A total of 931 spectra from seven ECC species were used to create the online database and included in the MSI software.

### Mass spectrometry systems for identification.

Two identification systems were used for the mass spectrum identification of isolates from the tested panel. The identifications were assigned an identification score. For the MALDI Biotyper v.4.1.90, 02/2020 (Bruker Daltonics), a log score of >2 is considered to indicate a high confidence level for identification at the species level, while for MSI, the threshold is a score of 20 (27).

Identification of interspecies discriminating peaks was performed with spectra from the online database (42 isolates) using the MALDIquant (24) and MALDIrrpa (28) packages in the R environment. The main discriminating peaks observed for the 7 ECC species (frequency greater than 95% for one species and absent for at least one species) were listed. An average spectrum containing aligned masses (tolerance = 150 ppm) and the average of intensities for each species was created to visualize peaks using the plotly package (29), available online at https://agodmer.github.io/ECC/.

### Statistical analysis of identifications.

The identification performances of the MSI and MALDI Biotyper systems were compared. The identification score and Cohen kappa coefficient were calculated using the caret package in the R environment (see Text S1 in the supplemental material for calculation details). The rate of correct identification from the tested panel by species from the three replicates was reported.

The confidence scores of the identifications generated by each of the two systems (MSI and MALDI Biotyper) associated with incorrect and correct identifications were compared with the nonparametric Mann-Whitney U test. From the confidence scores associated with the identification given by the two identification systems, receiver operating characteristic (ROC) curves were produced to evaluate the rates of correct and incorrect identifications as a function of the threshold scores using XLSTAT (v.2020.5.1.1042).
