# Supplementary material for: Revisiting Species Identification within the Enterobacter cloacae Complex by Matrix-Assisted Laser Desorption Ionization–Time of Flight Mass Spectrometry
Source: Microbiol Spectr. 2021 Aug 11;9(1):10.1128/spectrum.00661-21. doi: 10.1128/spectrum.00661-21 (PMC8552640; doi:10.1128/spectrum.00661-21)
Supplement: SUPPLEMENTAL FILE 1 — Supplemental material. Download SPECTRUM00661-21_Supp_1_seq1.pdf, PDF file, 0.7 MB [file spectrum00661-21_supp_1_seq1.pdf]

TEXT S1 Formulas used to calculate the correct identification rate and the associated precision

$$Percentage\ of\ correct\ identification = \frac{\sum correct\ identifications\ for\ a\ species}{\sum expected\ identifications\ \ for\ a\ species}$$

$$Precision = \frac{\sum correct\ identifications\ for\ a\ species}{\sum correct\ and\ incorrect\ identifications\ for\ a\ species}$$

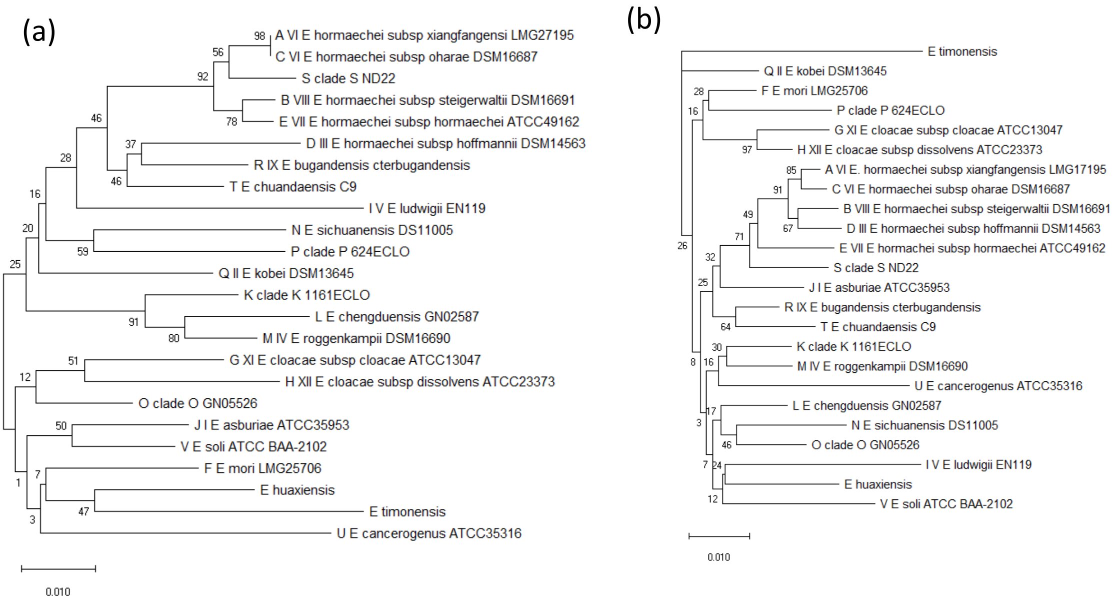

Notes: first letter is the first letter is the clade name according to Sutton (2) followed by the Roman numeral defined by Hofmann (1), then the species/subspecies and the isolate name.

(1). H. Hoffmann and A. Roggenkamp, “Population genetics of the nomenspecies *Enterobacter cloacae*,” *Appl. Environ. Microbiol.*, vol. 69, no. 9, pp. 5306–5318, Sep. 2003

(2) Sutton G.G., L. M. Brinkac L. M., Clarke T. H., Fouts D. E. **(2018)**, *Enterobacter hormaechei subsp. hoffmannii subsp. nov*., *Enterobacter hormaechei subsp. xiangfangensis comb. nov*., *Enterobacter roggenkampii sp. nov*., and *Enterobacter muelleri* is a later heterotypic synonym of *Enterobacter asburiae* based on computational a,” *F1000Research*, vol. 7

FIG S1 Phylogenetic trees obtained after sequencing of the *hsp60* gene. Left (a), tree constructed after sequencing from position 1218 to 1560 according to Hoffmann (1) and where *E. hormaechei* subspecies *xiangfangensis* and *oharae* are indistinguishable. Right (b), tree constructed according to the sequencing of position 230 to 1027 where subspecies *xiangfangensis* and *oharae* are discriminated. The evolutionary distances were computed using the Maximum Composite Likelihood method.

Table S1 Comparison of the identifications of 94 ECC strains obtained with the MSI system and the MALDI-biotyper system. Each strain was tested in triplica. The identification evaluation scores, i.e. the similarity score. For the MSI system and the log(score) for the Biotyper system are reported. The concordance between the expected identification and the identification given by the 2 systems is also reported.

		MSI identification				MALDI_Biotyper® identification				MSI identification		MALDI_Biotyper identification
name	hsp60 identification	similarity score 1	id 1	similarity score 2	id 2	Id 1	log(score) 1	Id 2	log(score) 2	concordance id 1 (0/1)	concordance id 2 (0/1)	concordance id1 (0/1)
3	E. hormaechei	66.14	E. hormaechei	53.74	E. hormaechei	E. cloacae	2.14	E. cloacae	2.13	1	1	0
3	E. hormaechei	62.81	E. hormaechei	49.81	E. hormaechei	E. cloacae	2.15	E. cloacae	2.1	1	1	0
3	E. hormaechei	69.44	E. hormaechei	58.84	E. hormaechei	E. cloacae	2.18	E. xiangfangensis	2.18	1	1	0
4	E. hormaechei	72.37	E. hormaechei	69.96	E. hormaechei	E. cloacae	2.24	E. xiangfangensis	2.16	1	1	0
4	E. hormaechei	55.98	E. hormaechei	54.52	E. hormaechei	E. hormaechei	2.26	E. cloacae	2.23	1	1	1
4	E. hormaechei	63.68	E. hormaechei	60.39	E. hormaechei	E. cloacae	2.3	E. xiangfangensis	2.29	1	1	0
6	E. hormaechei	58.56	E. hormaechei	57.9	E. hormaechei	E. hormaechei	2.27	E. cloacae	2.26	1	1	1
6	E. hormaechei	64.68	E. hormaechei	62.24	E. hormaechei	E. cloacae	2.28	E. xiangfangensis	2.18	1	1	0
6	E. hormaechei	65.14	E. hormaechei	60.47	E. hormaechei	E. hormaechei	2.3	E. cloacae	2.26	1	1	1
7	E. hormaechei	63.03	E. hormaechei	59.16	E. hormaechei	E. cloacae	2.23	E. cloacae	2.18	1	1	0
7	E. hormaechei	58.59	E. hormaechei	51.5	E. hormaechei	E. cloacae	2.3	E. cloacae	2.21	1	1	0
7	E. hormaechei	59.96	E. hormaechei	54.11	E. hormaechei	E. cloacae	2.31	E. cloacae	2.23	1	1	0
8	E. hormaechei	68.62	E. hormaechei	65.75	E. hormaechei	E. cloacae	2.22	E. cloacae	2.22	1	1	0
8	E. hormaechei	67.3	E. hormaechei	62.71	E. hormaechei	E. hormaechei	2.24	E. cloacae	2.21	1	1	1
8	E. hormaechei	65.78	E. hormaechei	60.38	E. hormaechei	E. hormaechei	2.28	E. cloacae	2.26	1	1	1
9	E. ludwigii	49.83	E. roggenkampii	45.56	E. bugandensis	E. cloacae	2.25	E. ludwigii	2.25	0	0	0
9	E. ludwigii	43.4	E. bugandensis	42.18	E. asburiae	E. cloacae	2.29	E. cloacae	2.09	0	0	0
9	E. ludwigii	51.39	E. ludwigii	42.51	E. roggenkampii	E. cloacae	2.3	E. ludwigii	2.2	1	0	0
10	E. hormaechei	60.7	E. hormaechei	58.83	E. hormaechei	E. cloacae	2.21	E. cloacae	2.19	1	1	0
10	E. hormaechei	54.15	E. hormaechei	52.64	E. hormaechei	E. cloacae	2.26	E. cloacae	2.14	1	1	0
10	E. hormaechei	42.08	E. hormaechei	40.4	E. hormaechei	E. cloacae	2.3	E. cloacae	2.11	1	1	0

11	E. hormaechei	53.48	E. hormaechei	51.58	E. hormaechei	E. cloacae	2.27	E. cloacae	2.2	1	1	0
11	E. hormaechei	62.48	E. hormaechei	57.53	E. hormaechei	E. cloacae	2.35	E. cloacae	2.24	1	1	0
11	E. hormaechei	48.5	E. hormaechei	47.87	E. hormaechei	E. cloacae	2.4	E. cloacae	2.29	1	1	0
12	E. hormaechei	57.84	E. hormaechei	54.87	E. hormaechei	E. cloacae	2.28	E. xiangfangensis	2.23	1	1	0
12	E. hormaechei	55.38	E. hormaechei	54.48	E. hormaechei	E. cloacae	2.36	E. cloacae	2.36	1	1	0
12	E. hormaechei	57.45	E. hormaechei	56.4	E. hormaechei	E. hormaechei	2.39	E. cloacae	2.23	1	1	1
13	E. hormaechei	57.04	E. hormaechei	53.34	E. hormaechei	E. cloacae	2.21	E. cloacae	2.16	1	1	0
13	E. hormaechei	55.53	E. hormaechei	54.29	E. hormaechei	E. cloacae	2.25	E. xiangfangensis	2.22	1	1	0
13	E. hormaechei	51.16	E. hormaechei	46.26	E. hormaechei	E. cloacae	2.27	E. cloacae	2.25	1	1	0
14	E. hormaechei	55.11	E. hormaechei	54.53	E. hormaechei	E. cloacae	2.2	E. cloacae	2.19	1	1	0
14	E. hormaechei	51.05	E. hormaechei	50.24	E. hormaechei	E. cloacae	2.23	E. cloacae	2.13	1	1	0
14	E. hormaechei	39.06	E. hormaechei	36.67	E. hormaechei	E. cloacae	2.35	E. cloacae	2.16	1	1	0
15	E. hormaechei	47.35	E. hormaechei	44.76	E. hormaechei	E. cloacae	2.26	E. cloacae	2.1	1	1	0
15	E. hormaechei	50.15	E. hormaechei	48.1	E. hormaechei	E. cloacae	2.29	E. cloacae	2.22	1	1	0
15	E. hormaechei	51.36	E. hormaechei	48.51	E. hormaechei	E. cloacae	2.31	E. xiangfangensis	2.24	1	1	0
16	E. hormaechei	54.51	E. hormaechei	44.48	E. hormaechei	E. cloacae	2.24	E. cloacae	2.16	1	1	0
16	E. hormaechei	49.04	E. hormaechei	43.3	E. hormaechei	E. hormaechei	2.27	E. cloacae	2.26	1	1	1
16	E. hormaechei	53.92	E. hormaechei	48.04	E. hormaechei	E. cloacae	2.28	E. cloacae	2.22	1	1	0
18	E. hormaechei	50.39	E. hormaechei	40.65	E. hormaechei	E. cloacae	2.19	E. cloacae	2.18	1	1	0
18	E. hormaechei	57.55	E. hormaechei	47.69	E. hormaechei	E. cloacae	2.25	E. cloacae	2.22	1	1	0
18	E. hormaechei	49.07	E. hormaechei	48.6	E. hormaechei	E. cloacae	2.31	E. cloacae	2.26	1	1	0
19	E. asburiae	56.98	E. asburiae	44.5	E. hormaechei	E. cloacae	2.15	E. asburiae	2.15	1	0	0
19	E. asburiae	57.16	E. asburiae	42.66	E. hormaechei	E. asburiae	2.19	E. asburiae	2.18	1	0	1
19	E. asburiae	57.83	E. asburiae	49.5	E. hormaechei	E. cloacae	2.25	E. asburiae	2.21	1	0	0
20	E. hormaechei	57.67	E. hormaechei	51.12	E. hormaechei	E. cloacae	2.21	E. cloacae	2.2	1	0	0
20	E. hormaechei	55.32	E. hormaechei	47.82	E. hormaechei	E. cloacae	2.22	E. cloacae	2.18	1	0	0
20	E. hormaechei	49.44	E. hormaechei	48.79	E. hormaechei	E. cloacae	2.23	E. cloacae	2.22	1	0	0
21	E. hormaechei	46.65	E. hormaechei	45.89	E. hormaechei	E. cloacae	2.22	E. cloacae	2.21	1	0	0
21	E. hormaechei	57.24	E. hormaechei	56.52	E. hormaechei	E. cloacae	2.23	E. cloacae	2.11	1	0	0
21	E. hormaechei	52.81	E. hormaechei	50.74	E. hormaechei	E. cloacae	2.23	E. cloacae	2.22	1	0	0
22	E. asburiae	50.84	E. asburiae	47.88	E. hormaechei	E. asburiae	2.16	E. cloacae	2.09	1	0	1
22	E. asburiae	55.33	E. asburiae	35.94	E. hormaechei	E. asburiae	2.17	E. bugandensis	2.17	1	0	1
22	E. asburiae	54.07	E. asburiae	49.14	E. hormaechei	E. bugandensis	2.19	E. asburiae	2.13	1	0	0

23	E. hormaechei	30.93	E. hormaechei	27.14	E. hormaechei	E. cloacae	2.22	E. cloacae	2.16	1	0	0
23	E. hormaechei	40.12	E. hormaechei	27.3	E. hormaechei	E. cloacae	2.22	E. cloacae	2.18	1	0	0
23	E. hormaechei	38.99	E. hormaechei	30.9	E. hormaechei	E. cloacae	2.25	E. cloacae	2.18	1	0	0
24	E. roggenkampii	56.81	E. roggenkampii	51.24	E. hormaechei	E. asburiae	2.12	E. cloacae	2.08	1	0	0
24	E. roggenkampii	72.31	E. roggenkampii	53.05	E. hormaechei	E. cloacae	2.17	E. kobei	2.03	1	0	0
24	E. roggenkampii	54.21	E. asburiae	53.78	E. hormaechei	E. asburiae	2.22	E. bugandensis	2.18	0	0	0
26	E. roggenkampii	58.47	E. roggenkampii	50.41	E. hormaechei	E. cloacae	2.17	E. cloacae	2.12	1	0	0
26	E. roggenkampii	50.76	E. asburiae	44.94	E. hormaechei	E. asburiae	2.19	E. bugandensis	2.14	0	0	0
26	E. roggenkampii	54.22	E. roggenkampii	50.5	E. hormaechei	E. asburiae	2.3	E. cloacae	2.13	1	0	0
27	E. hormaechei	47.03	E. hormaechei	46.78	E. hormaechei	E. cloacae	2.27	E. cloacae	2.19	1	0	0
27	E. hormaechei	44.36	E. hormaechei	42.74	E. hormaechei	E. cloacae	2.28	E. cloacae	2.26	1	0	0
27	E. hormaechei	43.78	E. hormaechei	42.45	E. hormaechei	E. cloacae	2.35	E. xiangfangensis	2.27	1	0	0
28	E. hormaechei	45.35	E. hormaechei	33.42	E. hormaechei	E. cloacae	2.17	E. cloacae	2.16	1	0	0
28	E. hormaechei	69.9	E. hormaechei	62.14	E. hormaechei	E. cloacae	2.21	E. cloacae	2.09	1	0	0
28	E. hormaechei	53.56	E. hormaechei	50.95	E. hormaechei	E. cloacae	2.27	E. cloacae	2.26	1	0	0
30	E. ludwigii	49.44	E. bugandensis	44.43	E. hormaechei	E. ludwigii	2.26	E. bugandensis	2.23	0	0	1
30	E. ludwigii	38.54	E. bugandensis	38.22	E. hormaechei	E. cloacae	2.28	E. ludwigii	2.19	0	0	0
30	E. ludwigii	45.05	E. bugandensis	43.46	E. hormaechei	E. ludwigii	2.31	E. cloacae	2.23	0	0	1
33	E. hormaechei	45.58	E. asburiae	43.7	E. hormaechei	E. cloacae	2.05	E. asburiae	2.04	0	0	0
33	E. hormaechei	42.9	E. asburiae	34.42	E. hormaechei	E. kobei	2.09	E. cloacae	2.08	0	0	0
33	E. hormaechei	50.26	E. asburiae	35.73	E. hormaechei	E. bugandensis	2.16	E. asburiae	2.13	0	0	0
34	E. hormaechei	50.02	E. hormaechei	46.24	E. hormaechei	E. cloacae	2.15	E. xiangfangensis	2.01	1	0	0
34	E. hormaechei	52.83	E. hormaechei	50.44	E. hormaechei	E. hormaechei	2.24	E. cloacae	2.23	1	0	1
34	E. hormaechei	57.15	E. hormaechei	56.17	E. hormaechei	E. cloacae	2.31	E. xiangfangensis	2.26	1	0	0
35	E. hormaechei	57.56	E. hormaechei	54.35	E. hormaechei	E. cloacae	2.19	E. cloacae	2.17	1	0	0
35	E. hormaechei	61.56	E. hormaechei	58.82	E. hormaechei	E. cloacae	2.2	E. cloacae	2.18	1	0	0
35	E. hormaechei	57.04	E. hormaechei	53.71	E. hormaechei	E. cloacae	2.26	E. cloacae	2.2	1	0	0
36	E. hormaechei	65.05	E. hormaechei	57.21	E. hormaechei	E. hormaechei	2.23	E. cloacae	2.22	1	0	1
36	E. hormaechei	53.95	E. hormaechei	53.75	E. hormaechei	E. hormaechei	2.25	E. cloacae	2.23	1	0	1
36	E. hormaechei	63.61	E. hormaechei	58.54	E. hormaechei	E. cloacae	2.27	E. cloacae	2.2	1	0	0
37	E. hormaechei	56.63	E. hormaechei	54.17	E. hormaechei	E. cloacae	2.17	E. cloacae	2.13	1	0	0
37	E. hormaechei	57.14	E. hormaechei	57.05	E. hormaechei	E. cloacae	2.18	E. cloacae	2.1	1	0	0
37	E. hormaechei	58.13	E. hormaechei	54.86	E. hormaechei	E. cloacae	2.25	E. cloacae	2.11	1	0	0

38	E. roggenkampii	41.9	E. kobei	41.2	E. hormaechei	E. asburiae	2.03	E. cloacae	1.98	0	0	0
38	E. roggenkampii	37.61	E. kobei	35.78	E. hormaechei	E. bugandensis	2.29	E. bugandensis	2.25	0	0	0
38	E. roggenkampii	58.4	E. kobei	57.35	E. hormaechei	E. bugandensis	2.35	E. bugandensis	2.34	0	0	0
40	E. roggenkampii	50.73	E. roggenkampii	39.52	E. hormaechei	E. asburiae	2.04	E. kobei	2.03	1	0	0
40	E. roggenkampii	53.68	E. roggenkampii	43.25	E. hormaechei	E. kobei	2.1	E. cloacae	2.08	1	0	0
40	E. roggenkampii	64.41	E. roggenkampii	50.05	E. hormaechei	E. cloacae	2.19	E. cloacae	2.18	1	0	0
41	E. roggenkampii	48.36	E. roggenkampii	47.42	E. hormaechei	E. cloacae	2.07	E. bugandensis	2.02	1	0	0
41	E. roggenkampii	49.43	E. roggenkampii	45.15	E. hormaechei	E. asburiae	2.13	E. cloacae	2.09	1	0	0
41	E. roggenkampii	54.86	E. roggenkampii	51.24	E. hormaechei	E. bugandensis	2.16	E. bugandensis	2.12	1	0	0
43	E. asburiae	54.15	E. asburiae	52.52	E. hormaechei	E. asburiae	2.05	E. bugandensis	2.05	1	0	1
43	E. asburiae	51.13	E. asburiae	46.58	E. hormaechei	E. bugandensis	2.05	E. bugandensis	2.02	1	0	0
43	E. asburiae	55.13	E. asburiae	53.13	E. hormaechei	E. asburiae	2.07	E. bugandensis	2	1	0	1
45	E. hormaechei	60.1	E. hormaechei	51.69	E. hormaechei	E. cloacae	2.22	E. cloacae	2.2	1	0	0
45	E. hormaechei	62.19	E. hormaechei	46.78	E. hormaechei	E. cloacae	2.27	E. xiangfangensis	2.23	1	0	0
45	E. hormaechei	69.97	E. hormaechei	55.45	E. hormaechei	E. cloacae	2.31	E. xiangfangensis	2.25	1	0	0
46	E. bugandensis	51.78	E. bugandensis	43.38	E. hormaechei	E. bugandensis	2.25	E. bugandensis	2.24	1	0	1
46	E. bugandensis	56.78	E. bugandensis	45.93	E. hormaechei	E. bugandensis	2.3	E. bugandensis	2.24	1	0	1
46	E. bugandensis	58.06	E. bugandensis	44.98	E. hormaechei	E. bugandensis	2.33	E. bugandensis	2.31	1	0	1
47	E. hormaechei	65.21	E. hormaechei	53.72	E. hormaechei	E. cloacae	2.22	E. cloacae	2.19	1	0	0
47	E. hormaechei	51.77	E. hormaechei	43.5	E. hormaechei	E. cloacae	2.28	E. cloacae	2.21	1	0	0
47	E. hormaechei	64.62	E. hormaechei	55.05	E. hormaechei	E. cloacae	2.29	E. xiangfangensis	2.21	1	0	0
48	E. hormaechei	59.17	E. hormaechei	50.33	E. hormaechei	E. cloacae	2.23	E. cloacae	2.22	1	0	0
48	E. hormaechei	57.04	E. hormaechei	51.82	E. hormaechei	E. cloacae	2.24	E. xiangfangensis	2.22	1	0	0
48	E. hormaechei	58.39	E. hormaechei	52.87	E. hormaechei	E. cloacae	2.24	E. cloacae	2.21	1	0	0
49	E. hormaechei	54.69	E. hormaechei	51.25	E. hormaechei	E. cloacae	2.18	E. cloacae	2.18	1	0	0
49	E. hormaechei	55.56	E. hormaechei	49.66	E. hormaechei	E. cloacae	2.26	E. cloacae	2.25	1	0	0
49	E. hormaechei	63.58	E. hormaechei	55.42	E. hormaechei	E. cloacae	2.26	E. cloacae	2.26	1	0	0
51	E. ludwigii	56.25	E. ludwigii	39.77	E. hormaechei	E. cloacae	2.26	E. ludwigii	2.18	1	0	0
51	E. ludwigii	61.32	E. ludwigii	47.62	E. hormaechei	E. cloacae	2.4	E. ludwigii	2.37	1	0	0
51	E. ludwigii	62.61	E. ludwigii	50.89	E. hormaechei	E. ludwigii	2.45	E. cloacae	2.42	1	0	1
52	E. hormaechei	32.22	E. hormaechei	31.92	E. hormaechei	E. cloacae	2.17	E. bugandensis	2.13	1	0	0
52	E. hormaechei	51.65	E. hormaechei	45.67	E. hormaechei	E. cloacae	2.2	E. cloacae	2.17	1	0	0
52	E. hormaechei	42.85	E. hormaechei	41.48	E. hormaechei	E. cloacae	2.27	E. cloacae	2.22	1	0	0

53	E. hormaechei	58.6	E. hormaechei	57.62	E. hormaechei	E. cloacae	2.32	E. cloacae	2.24	1	0	0
53	E. hormaechei	53.38	E. hormaechei	49.37	E. hormaechei	E. cloacae	2.33	E. cloacae	2.25	1	0	0
53	E. hormaechei	70.74	E. hormaechei	65.79	E. hormaechei	E. cloacae	2.38	E. cloacae	2.32	1	0	0
54	E. hormaechei	48.38	E. hormaechei	47.93	E. hormaechei	E. cloacae	2.2	E. xiangfangensis	2.17	1	0	0
54	E. hormaechei	46.36	E. hormaechei	42.49	E. hormaechei	E. hormaechei	2.26	E. cloacae	2.24	1	0	1
54	E. hormaechei	44.23	E. hormaechei	44.01	E. hormaechei	E. hormaechei	2.3	E. cloacae	2.27	1	0	1
55	E. hormaechei	53.01	E. hormaechei	43.9	E. hormaechei	E. cloacae	2.26	E. cloacae	2.18	1	0	0
55	E. hormaechei	50.47	E. hormaechei	47.07	E. hormaechei	E. cloacae	2.26	E. cloacae	2.15	1	0	0
55	E. hormaechei	77.14	E. hormaechei	65.73	E. hormaechei	E. cloacae	2.45	E. cloacae	2.33	1	0	0
56	E. hormaechei	51.93	E. hormaechei	50.13	E. hormaechei	E. cloacae	2.26	E. cloacae	2.25	1	0	0
56	E. hormaechei	56.99	E. hormaechei	53.42	E. hormaechei	E. hormaechei	2.33	E. cloacae	2.33	1	0	1
56	E. hormaechei	65.38	E. hormaechei	64.51	E. hormaechei	E. cloacae	2.43	E. cloacae	2.41	1	0	0
57	E. roggenkampii	57.22	E. roggenkampii	46.91	E. hormaechei	E. kobei	2.25	E. asburiae	2.19	1	0	0
57	E. roggenkampii	60.32	E. roggenkampii	51.07	E. hormaechei	E. kobei	2.25	E. bugandensis	2.22	1	0	0
57	E. roggenkampii	55.4	E. roggenkampii	49.95	E. hormaechei	E. kobei	2.28	E. asburiae	2.25	1	0	0
58	E. hormaechei	50	E. hormaechei	42.54	E. hormaechei	E. cloacae	2.18	E. bugandensis	2.11	1	0	0
58	E. hormaechei	55.25	E. hormaechei	47.65	E. hormaechei	E. hormaechei	2.25	E. cloacae	2.23	1	0	1
58	E. hormaechei	68.01	E. hormaechei	55.36	E. hormaechei	E. cloacae	2.42	E. cloacae	2.29	1	0	0
60	E. hormaechei	46.68	E. hormaechei	45.45	E. hormaechei	E. hormaechei	2.24	E. bugandensis	2.16	1	0	1
60	E. hormaechei	66.08	E. hormaechei	64.17	E. hormaechei	E. cloacae	2.36	E. xiangfangensis	2.24	1	0	0
60	E. hormaechei	64.6	E. hormaechei	61.89	E. hormaechei	E. hormaechei	2.41	E. cloacae	2.4	1	0	1
61	E. hormaechei	67.01	E. hormaechei	53.5	E. hormaechei	E. cloacae	2.36	E. xiangfangensis	2.27	1	0	0
61	E. hormaechei	67.57	E. hormaechei	57.64	E. hormaechei	E. cloacae	2.38	E. cloacae	2.25	1	0	0
61	E. hormaechei	59.81	E. hormaechei	55.24	E. hormaechei	E. cloacae	2.44	E. cloacae	2.33	1	0	0
62	E. hormaechei	43.68	E. hormaechei	41.96	E. hormaechei	E. cloacae	2.04	E. cloacae	2	1	0	0
62	E. hormaechei	59.04	E. hormaechei	56.94	E. hormaechei	E. cloacae	2.12	E. cloacae	2.1	1	0	0
62	E. hormaechei	59.63	E. hormaechei	52.91	E. hormaechei	E. cloacae	2.34	E. xiangfangensis	2.29	1	0	0
64	E. hormaechei	44.37	E. hormaechei	43.19	E. hormaechei	E. cloacae	2.28	E. cloacae	2.2	1	0	0
64	E. hormaechei	66.57	E. hormaechei	54.12	E. hormaechei	E. cloacae	2.37	E. xiangfangensis	2.22	1	0	0
64	E. hormaechei	70.31	E. hormaechei	57.06	E. hormaechei	E. cloacae	2.4	E. cloacae	2.32	1	0	0
65	E. hormaechei	45.31	E. hormaechei	39.25	E. hormaechei	E. cloacae	2.14	E. cloacae	2.1	1	0	0
65	E. hormaechei	63.79	E. hormaechei	62.28	E. hormaechei	E. cloacae	2.24	E. cloacae	2.1	1	0	0
65	E. hormaechei	67.72	E. hormaechei	58.64	E. hormaechei	E. cloacae	2.28	E. cloacae	2.26	1	0	0

66	E. bugandensis	58.84	E. bugandensis	58.69	E. hormaechei	E. bugandensis	2.28	E. bugandensis	2.22	1	0	1
66	E. bugandensis	44.37	E. bugandensis	43.76	E. hormaechei	E. bugandensis	2.39	E. bugandensis	2.36	1	0	1
66	E. bugandensis	55.88	E. bugandensis	53.99	E. hormaechei	E. bugandensis	2.42	E. bugandensis	2.27	1	0	1
68	E. hormaechei	62.36	E. hormaechei	60.59	E. hormaechei	E. cloacae	2.25	E. cloacae	2.16	1	0	0
68	E. hormaechei	56.82	E. hormaechei	53.97	E. hormaechei	E. cloacae	2.31	E. xiangfangensis	2.25	1	0	0
68	E. hormaechei	67.78	E. hormaechei	63.22	E. hormaechei	E. cloacae	2.35	E. xiangfangensis	2.23	1	0	0
69	E. roggenkampii	61.9	E. ludwigii	45.48	E. hormaechei	E. ludwigii	2.37	E. cloacae	2.27	0	0	0
69	E. roggenkampii	65.88	E. ludwigii	46.07	E. hormaechei	E. ludwigii	2.37	E. cloacae	2.27	0	0	0
69	E. roggenkampii	63.77	E. ludwigii	44.87	E. hormaechei	E. ludwigii	2.38	E. cloacae	2.29	0	0	0
70	E. ludwigii	57.97	E. ludwigii	44.61	E. hormaechei	E. ludwigii	2.17	E. bugandensis	2.14	1	0	1
70	E. ludwigii	38.13	E. ludwigii	32.15	E. hormaechei	E. ludwigii	2.29	E. asburiae	2.11	1	0	1
70	E. ludwigii	50.42	E. ludwigii	39.81	E. hormaechei	E. ludwigii	2.33	E. cloacae	2.23	1	0	1
72	E. hormaechei	52.11	E. hormaechei	50.69	E. hormaechei	E. cloacae	2.26	E. xiangfangensis	2.26	1	0	0
72	E. hormaechei	57.66	E. hormaechei	52.09	E. hormaechei	E. cloacae	2.28	E. xiangfangensis	2.23	1	0	0
72	E. hormaechei	53.52	E. hormaechei	49.87	E. hormaechei	E. cloacae	2.29	E. xiangfangensis	2.24	1	0	0
73	E. asburiae	59.33	E. asburiae	57.58	E. hormaechei	E. bugandensis	2.25	E. bugandensis	2.24	1	0	0
73	E. asburiae	61.73	E. asburiae	58.23	E. hormaechei	E. bugandensis	2.33	E. asburiae	2.32	1	0	0
73	E. asburiae	62.76	E. asburiae	56.13	E. hormaechei	E. bugandensis	2.35	E. bugandensis	2.33	1	0	0
74	E. asburiae	56.23	E. kobei	56.08	E. hormaechei	E. asburiae	2.1	E. bugandensis	2.08	0	0	1
74	E. asburiae	50.32	E. asburiae	47.89	E. hormaechei	E. asburiae	2.2	E. asburiae	2.14	1	0	1
74	E. asburiae	54.39	E. asburiae	51.97	E. hormaechei	E. asburiae	2.25	E. bugandensis	2.24	1	0	1
75	E. hormaechei	63.37	E. hormaechei	57.37	E. hormaechei	E. hormaechei	2.25	E. cloacae	2.19	1	0	1
75	E. hormaechei	54.75	E. hormaechei	50.12	E. hormaechei	E. hormaechei	2.26	E. cloacae	2.18	1	0	1
75	E. hormaechei	56.99	E. hormaechei	49.33	E. hormaechei	E. hormaechei	2.29	E. cloacae	2.24	1	0	1
76	E. hormaechei	61.87	E. hormaechei	60.46	E. hormaechei	E. cloacae	2.14	E. cloacae	2.13	1	0	0
76	E. hormaechei	56.5	E. hormaechei	56.06	E. hormaechei	E. cloacae	2.33	E. cloacae	2.29	1	0	0
76	E. hormaechei	60.89	E. hormaechei	59.03	E. hormaechei	E. hormaechei	2.37	E. cloacae	2.34	1	0	1
81	E. hormaechei	35.65	E. hormaechei	31.91	E. hormaechei	E. cloacae	2.08	E. cloacae	2.01	1	0	0
81	E. hormaechei	64.26	E. hormaechei	53.05	E. hormaechei	E. cloacae	2.14	E. cloacae	2.09	1	0	0
81	E. hormaechei	43.34	E. hormaechei	42.29	E. hormaechei	E. cloacae	2.14	E. cloacae	2.14	1	0	0
86	E. hormaechei	63.02	E. hormaechei	51	E. hormaechei	E. cloacae	2.15	E. cloacae	2.08	1	0	0
86	E. hormaechei	61.62	E. hormaechei	49.41	E. hormaechei	E. hormaechei	2.28	E. cloacae	2.22	1	0	1
86	E. hormaechei	54.52	E. hormaechei	54.25	E. hormaechei	E. hormaechei	2.33	E. cloacae	2.28	1	0	1

88	E. hormaechei	55.54	E. hormaechei	48.36	E. hormaechei	E. cloacae	2.16	E. xiangfangensis	2.16	1	0	0
88	E. hormaechei	44.84	E. hormaechei	44.58	E. hormaechei	E. cloacae	2.2	E. xiangfangensis	2.08	1	0	0
88	E. hormaechei	48.56	E. hormaechei	40.99	E. hormaechei	E. cloacae	2.21	E. xiangfangensis	2.08	1	0	0
89	E. hormaechei	42.12	E. hormaechei	35.65	E. hormaechei	E. cloacae	2.13	E. cloacae	2.05	1	0	0
89	E. hormaechei	47.81	E. hormaechei	47.43	E. hormaechei	E. cloacae	2.27	E. xiangfangensis	2.21	1	0	0
89	E. hormaechei	55.39	E. hormaechei	53.05	E. hormaechei	E. cloacae	2.27	E. xiangfangensis	2.13	1	0	0
90	E. hormaechei	47.1	E. hormaechei	42.01	E. hormaechei	E. hormaechei	2.19	E. cloacae	2.09	1	0	1
90	E. hormaechei	47.23	E. kobei	44.36	E. hormaechei	E. hormaechei	2.21	E. cloacae	2.19	0	0	1
90	E. hormaechei	45.98	E. hormaechei	42.79	E. hormaechei	E. cloacae	2.25	E. xiangfangensis	2.22	1	0	0
91	E. hormaechei	59.92	E. hormaechei	54.63	E. hormaechei	E. cloacae	2.19	E. cloacae	2.17	1	0	0
91	E. hormaechei	54.93	E. hormaechei	46.17	E. hormaechei	E. cloacae	2.2	E. cloacae	2.19	1	0	0
91	E. hormaechei	63.22	E. hormaechei	54.39	E. hormaechei	E. cloacae	2.24	E. cloacae	2.22	1	0	0
92	E. bugandensis	51.88	E. kobei	48.48	E. hormaechei	E. bugandensis	2.16	E. bugandensis	2.16	0	0	1
92	E. bugandensis	58.8	E. bugandensis	56.33	E. hormaechei	E. bugandensis	2.36	E. bugandensis	2.35	1	0	1
92	E. bugandensis	57.39	E. kobei	53.18	E. hormaechei	E. bugandensis	2.38	E. bugandensis	2.35	0	0	1
95	E. hormaechei	62.36	E. hormaechei	51.32	E. hormaechei	E. cloacae	2	E. cloacae	1.92	1	0	0
95	E. hormaechei	50.55	E. hormaechei	47.36	E. hormaechei	E. cloacae	2.3	E. xiangfangensis	2.23	1	0	0
95	E. hormaechei	47.97	E. bugandensis	44.37	E. hormaechei	E. bugandensis	2.47	E. bugandensis	2.41	0	0	0
108	E. hormaechei	61.57	E. hormaechei	58.15	E. hormaechei	E. hormaechei	2.22	E. cloacae	2.17	1	0	1
108	E. hormaechei	63.58	E. hormaechei	59.07	E. hormaechei	E. cloacae	2.27	E. xiangfangensis	2.2	1	0	0
108	E. hormaechei	55.32	E. hormaechei	50.46	E. hormaechei	E. hormaechei	2.27	E. cloacae	2.21	1	0	1
110	E. hormaechei	56.17	E. hormaechei	48.42	E. hormaechei	E. hormaechei	2.21	E. cloacae	2.15	1	0	1
110	E. hormaechei	60.73	E. hormaechei	57.22	E. hormaechei	E. cloacae	2.22	E. xiangfangensis	2.18	1	0	0
110	E. hormaechei	68.85	E. hormaechei	59.49	E. hormaechei	E. hormaechei	2.22	E. cloacae	2.14	1	0	1
111	E. hormaechei	63	E. hormaechei	57.35	E. hormaechei	E. hormaechei	2.19	E. cloacae	2.17	1	0	1
111	E. hormaechei	63.26	E. hormaechei	58.44	E. hormaechei	E. cloacae	2.24	E. xiangfangensis	2.23	1	0	0
111	E. hormaechei	53.07	E. hormaechei	48.29	E. hormaechei	E. hormaechei	2.24	E. cloacae	2.19	1	0	1
131	E. ludwigii	62.39	E. ludwigii	49.9	E. hormaechei	E. cloacae	2.27	E. ludwigii	2.25	1	0	0
131	E. ludwigii	55.57	E. ludwigii	44.89	E. hormaechei	E. ludwigii	2.3	E. cloacae	2.23	1	0	1
131	E. ludwigii	62.79	E. ludwigii	50.24	E. hormaechei	E. cloacae	2.31	E. ludwigii	2.25	1	0	0
153	E. roggenkampii	55.84	E. roggenkampii	51.86	E. hormaechei	E. bugandensis	2.22	E. asburiae	2.22	1	0	0
153	E. roggenkampii	55.73	E. roggenkampii	53.27	E. hormaechei	E. bugandensis	2.23	E. bugandensis	2.16	1	0	0
153	E. roggenkampii	65.8	E. roggenkampii	56.52	E. hormaechei	E. kobei	2.28	E. asburiae	2.27	1	0	0

186	E. cloacae	56.04	E. cloacae	50.41	E. hormaechei	E. bugandensis	2.2	E. bugandensis	2.19	1	0	0
186	E. cloacae	44.96	E. kobei	44.15	E. hormaechei	E. bugandensis	2.25	E. bugandensis	2.24	0	0	0
186	E. cloacae	38.42	E. cloacae	37.21	E. hormaechei	E. asburiae	2.38	E. bugandensis	2.25	1	0	0
265	E. asburiae	60.29	E. asburiae	60.21	E. hormaechei	E. cloacae	2.29	E. cloacae	2.26	1	0	0
265	E. asburiae	66.58	E. asburiae	61.16	E. hormaechei	E. cloacae	2.32	E. cloacae	2.26	1	0	0
265	E. asburiae	61.47	E. asburiae	54.29	E. hormaechei	E. cloacae	2.32	E. cloacae	2.3	1	0	0
322	E. ludwigii	55.58	E. ludwigii	49.93	E. hormaechei	E. ludwigii	2.28	E. bugandensis	2.26	1	0	1
322	E. ludwigii	59.04	E. ludwigii	51.52	E. hormaechei	E. ludwigii	2.3	E. bugandensis	2.12	1	0	1
322	E. ludwigii	55.99	E. ludwigii	45.35	E. hormaechei	E. ludwigii	2.35	E. bugandensis	2.26	1	0	1
506	E. hormaechei	62.44	E. hormaechei	49.25	E. hormaechei	E. cloacae	2.16	E. cloacae	2.15	1	0	0
506	E. hormaechei	68.35	E. hormaechei	60.12	E. hormaechei	E. hormaechei	2.2	E. cloacae	2.16	1	0	1
506	E. hormaechei	68.37	E. hormaechei	54.33	E. hormaechei	E. cloacae	2.28	E. cloacae	2.22	1	0	0
612	E. hormaechei	67.84	E. hormaechei	59.83	E. hormaechei	E. cloacae	2.2	E. cloacae	2.04	1	0	0
612	E. hormaechei	81.35	E. hormaechei	68	E. hormaechei	E. cloacae	2.21	E. xiangfangensis	2.18	1	0	0
612	E. hormaechei	56.03	E. hormaechei	53.76	E. hormaechei	E. cloacae	2.27	E. xiangfangensis	2.19	1	0	0
618	E. kobei	63.75	E. kobei	59.19	E. hormaechei	E. bugandensis	2.12	E. bugandensis	2.09	1	0	0
618	E. kobei	53.13	E. kobei	50.79	E. hormaechei	E. bugandensis	2.2	E. bugandensis	2.15	1	0	0
618	E. kobei	62.79	E. kobei	58.26	E. hormaechei	E. bugandensis	2.27	E. bugandensis	2.25	1	0	0
619	E. kobei	56.24	E. kobei	47.57	E. hormaechei	E. bugandensis	2.2	E. bugandensis	2.18	1	0	0
619	E. kobei	68.78	E. kobei	59.54	E. hormaechei	E. bugandensis	2.31	E. bugandensis	2.29	1	0	0
621	E. hormaechei	67.64	E. hormaechei	56	E. hormaechei	E. cloacae	2.24	E. cloacae	2.21	1	0	0
621	E. hormaechei	66.84	E. hormaechei	54.92	E. hormaechei	E. cloacae	2.25	E. cloacae	2.21	1	0	0
621	E. hormaechei	61.9	E. hormaechei	50.82	E. hormaechei	E. cloacae	2.3	E. cloacae	2.24	1	0	0
710	E. hormaechei	75.48	E. hormaechei	66.76	E. hormaechei	E. cloacae	2.15	E. asburiae	2.02	1	0	0
710	E. hormaechei	76.34	E. hormaechei	70.06	E. hormaechei	E. cloacae	2.23	E. cloacae	2.02	1	0	0
710	E. hormaechei	80.05	E. hormaechei	68.16	E. hormaechei	E. cloacae	2.25	E. cloacae	2.11	1	0	0
714	E. hormaechei	59.31	E. hormaechei	55.92	E. hormaechei	E. cloacae	2.19	E. xiangfangensis	2.16	1	0	0
714	E. hormaechei	55.4	E. hormaechei	51.65	E. hormaechei	E. cloacae	2.27	E. asburiae	2.18	1	0	0
714	E. hormaechei	58.93	E. hormaechei	58.11	E. hormaechei	E. cloacae	2.28	E. cloacae	2.22	1	0	0
716	E. hormaechei	58.32	E. hormaechei	55.5	E. hormaechei	E. cloacae	2.32	E. xiangfangensis	2.21	1	0	0
716	E. hormaechei	66.13	E. hormaechei	56.13	E. hormaechei	E. cloacae	2.32	E. xiangfangensis	2.15	1	0	0
716	E. hormaechei	64.63	E. hormaechei	55.89	E. hormaechei	E. cloacae	2.4	E. cloacae	2.24	1	0	0
725	E. hormaechei	54.44	E. hormaechei	52.14	E. hormaechei	E. cloacae	2.21	E. cloacae	2.14	1	0	0

725	E. hormaechei	63.87	E. hormaechei	60.74	E. hormaechei	E. cloacae	2.31	E. cloacae	2.31	1	0	0
725	E. hormaechei	54.62	E. hormaechei	52.67	E. hormaechei	E. cloacae	2.32	E. cloacae	2.31	1	0	0
731	E. hormaechei	54.15	E. hormaechei	52.43	E. hormaechei	E. hormaechei	2.31	E. cloacae	2.24	1	0	1
731	E. hormaechei	67.78	E. hormaechei	61.48	E. hormaechei	E. hormaechei	2.31	E. cloacae	2.2	1	0	1
731	E. hormaechei	60.23	E. hormaechei	58.59	E. hormaechei	E. cloacae	2.34	E. xiangfangensis	2.29	1	0	0
906	E. hormaechei	70.25	E. hormaechei	61.32	E. hormaechei	E. hormaechei	2.17	E. cloacae	2.16	1	0	1
906	E. hormaechei	57.83	E. hormaechei	51.82	E. hormaechei	E. cloacae	2.23	E. asburiae	2.1	1	0	0
906	E. hormaechei	59.96	E. hormaechei	53.38	E. hormaechei	E. cloacae	2.28	E. cloacae	2.2	1	0	0
907	E. hormaechei	54.29	E. hormaechei	50.24	E. hormaechei	E. hormaechei	2.24	E. cloacae	2.23	1	0	1
907	E. hormaechei	74.28	E. hormaechei	64.88	E. hormaechei	E. cloacae	2.25	E. cloacae	2.16	1	0	0
907	E. hormaechei	59.58	E. hormaechei	54.76	E. hormaechei	E. cloacae	2.28	E. xiangfangensis	2.24	1	0	0
908	E. hormaechei	56.29	E. hormaechei	54.16	E. hormaechei	E. hormaechei	2.24	E. cloacae	2.23	1	0	1
908	E. hormaechei	54.86	E. hormaechei	53.96	E. hormaechei	E. cloacae	2.25	E. xiangfangensis	2.24	1	0	0
908	E. hormaechei	55.95	E. hormaechei	55.86	E. hormaechei	E. hormaechei	2.29	E. cloacae	2.29	1	0	1
915	E. hormaechei	64.29	E. hormaechei	52.72	E. hormaechei	E. cloacae	2.26	E. cloacae	2.22	1	0	0
915	E. hormaechei	58.74	E. hormaechei	57.74	E. hormaechei	E. hormaechei	2.3	E. cloacae	2.29	1	0	1
915	E. hormaechei	60.12	E. hormaechei	57.98	E. hormaechei	E. cloacae	2.33	E. cloacae	2.32	1	0	0
917	E. kobei	61.53	E. kobei	54.74	E. hormaechei	E. bugandensis	2.24	E. kobei	2.23	1	0	0
917	E. kobei	56.02	E. kobei	52.72	E. hormaechei	E. kobei	2.28	E. bugandensis	2.2	1	0	1
917	E. kobei	61.7	E. kobei	60.09	E. hormaechei	E. kobei	2.31	E. bugandensis	2.29	1	0	1
918	E. hormaechei	75.88	E. hormaechei	67.05	E. hormaechei	E. cloacae	2.27	E. cloacae	2.1	1	1	0
918	E. hormaechei	58.11	E. hormaechei	53.43	E. hormaechei	E. cloacae	2.3	E. xiangfangensis	2.3	1	1	0
918	E. hormaechei	74.17	E. hormaechei	64.92	E. hormaechei	E. cloacae	2.34	E. asburiae	2.17	1	1	0
919	E. hormaechei	49.74	E. hormaechei	46.66	E. hormaechei	E. hormaechei	2.12	E. cloacae	2.11	1	1	1
919	E. hormaechei	54.35	E. hormaechei	51.26	E. hormaechei	E. cloacae	2.12	E. hormaechei	2.12	1	1	0
919	E. hormaechei	56.12	E. hormaechei	50.4	E. hormaechei	E. cloacae	2.16	E. cloacae	2.13	1	1	0
96	E. cloacae	46.61	E. cloacae	42.78	E. kobei	E. cloacae	2.28	E. cloacae	2.18	1	0	1
96	E. cloacae	50.01	E. cloacae	43.6	E. kobei	E. cloacae	2.3	E. cloacae	2.27	1	0	1
96	E. cloacae	39.56	E. cloacae	36.09	E. kobei	E. cloacae	2.3	E. cloacae	2.28	1	0	1

TABLE S2 Main discriminating peaks observed for the 7 species of *Enterobacter sp.*

(frequency greater than 95% for one species and absent for at least one species)

	Discriminant peaks	*E. asburiae*	*E. bugandensis*	*E. cloacae*	*E. hormaechei*	*E. kobei*	*E. ludwigii*	*E. roggenkampii*
E. asburiae	2581,57	97,5	100		0,4	11,3	13,3	63,9
	3545,98	100	100	1,5	17,4	23,8	100	
	5167,71	100	100		0,2	75		59
	6376,64	98,7	100		71	97,5	6,7	100
	7058,56	100	94,7	100	3		97,8	
	7092,75	100	100	10,8	3		100	1,6
	12625,76	97,5	93,3		61,3	86,3	46,7	0,8

	Discriminant peaks	*E. asburiae*	*E. bugandensis*	*E. cloacae*	*E. hormaechei*	*E. kobei*	*E. ludwigii*	*E. roggenkampii*
E. bugandensis	2581,57	97,5	100		0,4	11,3	13,3	63,9
	3075,39	53,2	100		7,1	37,5	4,4	29,5
	3545,98	100	100	1,5	17,4	23,8	100	
	3733,37	60,8	100		76,8	63,8	71,1	84,4
	4141,02	19	97,3		64,1	98,8	28,9	45,1
	5167,71	100	100		0,2	75		59
	5645,13		100	23,1	94,8	48,8	100	
	6154,27	82,3	100	64,6	1,9	67,5		62,3
	6376,64	98,7	100		71	97,5	6,7	100
	7092,75	100	100	10,8	3		100	1,6
	10997,45	44,3	100	98,5	20		64,4	

	Discriminant peaks	*E. asburiae*	*E. bugandensis*	*E. cloacae*	*E. hormaechei*	*E. kobei*	*E. ludwigii*	*E. roggenkampii*
E. cloacae	3082,84	11,4		100	0,9		17,8	
	3327,83	3,8		100	0,9	2,5	24,4	
	3740,14	5,1		100	12,9	6,3	4,4	13,9
	4210,88		73,3	100	38,5			
	5121,17	10,1		100	50,1		100	5,7
	5294,46	30,4	69,3	98,5	9,2	22,5		4,1
	5611,11			100	21,1	3,8		40,2
	5837,59	74,7		95,4	12,5	45	48,9	100
	6427,54	29,1	4	96,9	28,2		8,9	27
	7058,56	100	94,7	100	3		97,8	
	7722,19	7,6		100	69	3,8		59,8
	10850,66	13,9	30,7	95,4	55,7		73,3	15,6
	10997,45	44,3	100	98,5	20		64,4	
	12980,78		93,3	100				

	Discriminant peaks	*E. asburiae*	*E. bugandensis*	*E. cloacae*	*E. hormaechei*	*E. kobei*	*E. ludwigii*	*E. roggenkampii*
E. hormaechei	3844,93	1,3	13,3	30,8	95,7	6,3	2,2	
	8517,38	16,5			96,3	5	33,3	31,1
	8997,67			9,2	100			

	Discriminant peaks	*E. asburiae*	*E. bugandensis*	*E. cloacae*	*E. hormaechei*	*E. kobei*	*E. ludwigii*	*E. roggenkampii*
E. kobei	4141,02	19	97,3		64,1	98,8	28,9	45,1
	6376,64	98,7	100		71	97,5	6,7	100
	7321,39	38	52	83,1	64,3	100		86,9
	9589,49	84,8	54,7	18,5	54,4	100		10,7

	Discriminant peaks	*E. asburiae*	*E. bugandensis*	*E. cloacae*	*E. hormaechei*	*E. kobei*	*E. ludwigii*	*E. roggenkampii*
E. ludwigii	3501,28	57	70,7	18,5	28,6	18,8	95,6	
	3545,98	100	100	1,5	17,4	23,8	100	
	5121,17	10,1		100	50,1		100	5,7
	5645,13		100	23,1	94,8	48,8	100	
	6361,75	49,4	8	86,2	19,1	18,8	100	
	6646,7		4		3,2		97,8	
	7058,56	100	94,7	100	3		97,8	
	7092,75	100	100	10,8	3		100	1,6
	7225,23	17,7		60		26,3	97,8	34,4
	7308,64	20,3	20	20	49,9		100	58,2

	Discriminant peaks	*E. asburiae*	*E. bugandensis*	*E. cloacae*	*E. hormaechei*	*E. kobei*	*E. ludwigii*	*E. roggenkampii*
E. roggenkampii	3599,69	59,5	54,7	35,4	26,5	33,8		98,4
	4121,7	81		29,2	10,8	1,3		100
	4218,67				4,3			99,2
	5440,03	67,1	89,3	24,6	37,8	88,8		95,9
	5837,59	74,7		95,4	12,5	45	48,9	100
	6376,64	98,7	100		71	97,5	6,7	100
	7202,9	75,9	38,7	36,9	19,6	12,5		100
	8240,46	81	33,3	35,4		78,8		100
	9080,58	5,1		49,2	22,6	41,3		100
	12655,79			73,8	6,2	2,5	8,9	100

Notes: in red, frequencies ≥95%; Dark orange, frequencies ∈ [50,95[; Light orange, frequencies ∈ [20,50[; Yellow, frequencies ∈ [10, 20[; Light green, frequencies ∈ [5, 10[; Dark green, frequencies ∈]0,5[.

FIG S2 Receiver Operating Characteristic (ROC) analysis for Bruker system.

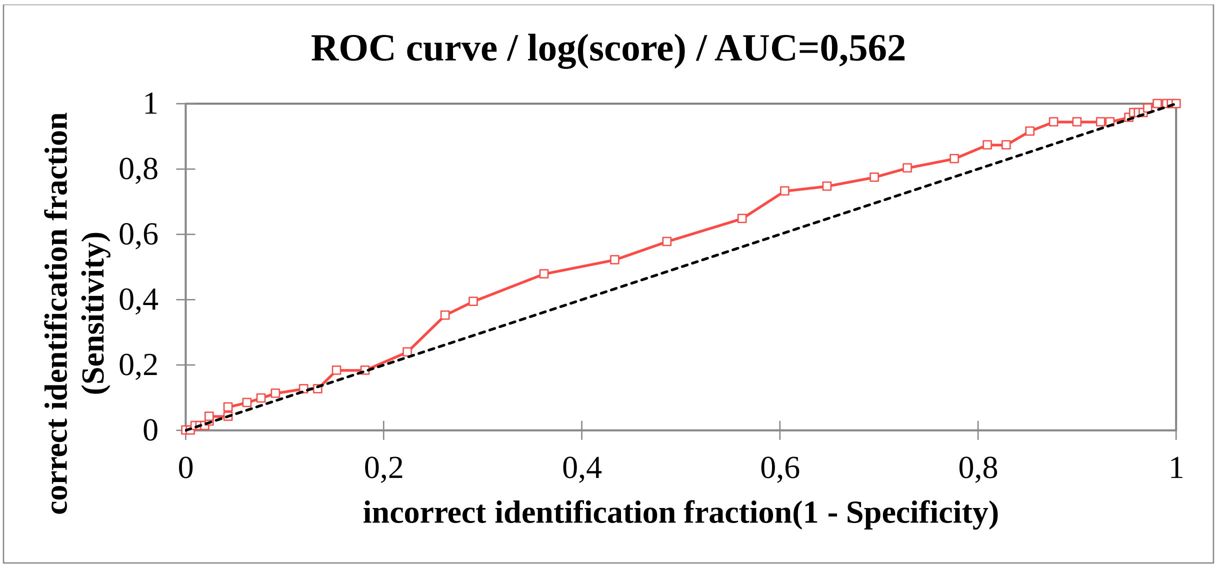

Note : Area Under Curve (AUC) is equal to 0.56 and not significantly different from 0.50 (Z-test, *p* >0.05)

FIG S3 Receiver Operating Characteristic (ROC) analysis for MSI system

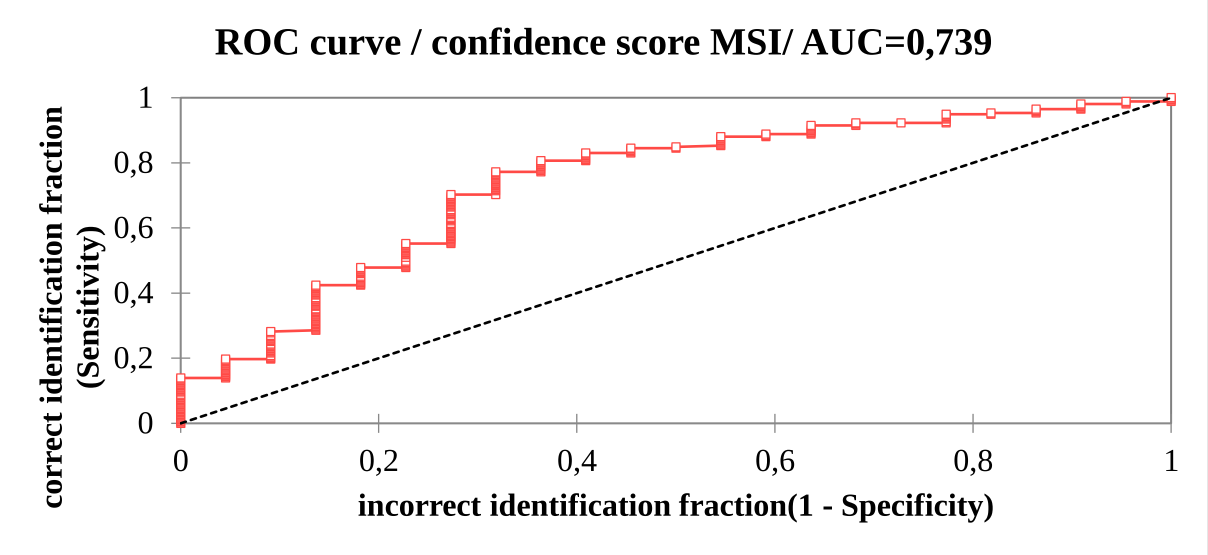

Note : Area Under Curve (AUC) is equal to 0.74 and significantly different from 0.50 (Z-test, $p < 0,0001$)
